# Changes in Vowel Articulation with Subthalamic Nucleus Deep Brain Stimulation in Dysarthric Speakers with Parkinson's Disease

**DOI:** 10.1155/2014/487035

**Published:** 2014-10-21

**Authors:** Vincent Martel Sauvageau, Joël Macoir, Mélanie Langlois, Michel Prud'Homme, Léo Cantin, Johanna-Pascale Roy

**Affiliations:** ^1^Centre de Recherche, Institut Universitaire en Santé Mentale de Québec, Laboratoire Langage et Cognition, Québec, QC, Canada G1J 2G3; ^2^CHU de Québec-Enfant-Jésus, Département des Sciences Neurologiques, Québec, QC, Canada G1J 1Z4; ^3^Université Laval, Centre Interdisciplinaire de Recherche sur Les Activités Langagières, Québec, QC, Canada G1V 0A6

## Abstract

*Purpose*. To investigate changes in vowel articulation with the electrical deep brain stimulation (DBS) of the subthalamic nucleus (STN) in dysarthric speakers with Parkinson's disease (PD). *Methods*. Eight Quebec-French speakers diagnosed with idiopathic PD who had undergone STN DBS were evaluated ON-stimulation and OFF-stimulation (1 hour after DBS was turned off). Vowel articulation was compared ON-simulation versus OFF-stimulation using acoustic vowel space and formant centralization ratio, calculated with the first (*F*1) and second formant (*F*2) of the vowels /i/, /u/, and /a/. The impact of the preceding consonant context on articulation, which represents a measure of coarticulation, was also analyzed as a function of the stimulation state. *Results*. Maximum vowel articulation increased during ON-stimulation. Analyses also indicate that vowel articulation was modulated by the consonant context but this relationship did not change with STN DBS. *Conclusions*. Results suggest that STN DBS may improve articulation in dysarthric speakers with PD, in terms of range of movement. Optimization of the electrical parameters for each patient is important and may lead to improvement in speech fine motor control. However, the impact on overall speech intelligibility may still be small. Clinical considerations are discussed and new research avenues are suggested.

## 1. Introduction

Parkinson's disease (PD) has traditionally been considered strictly as a motor disorder with symptoms such as tremor, muscle rigidity, and bradykinesia. In recent decades, many researchers have investigated other clinical manifestations of PD, such as mood changes, language impairment, cognition, and sleep disorders. Today, PD is commonly viewed as a multisystemic degenerative disorder [1]. Alongside these symptoms, up to 90% of people with PD develop speech disorders over the course of the disease [2]. In many studies, authors investigated the impairment of speech systems in PD from a motor, acoustic, or perceptual point of view. Studies examining physiological changes in the speech systems of people with PD reported impaired respiratory [3], laryngeal [4], and orofacial [5, 6] functions which have an impact on the acoustic signal of speech. Studies investigating such changes reported reduced intensity level [7] and fundamental frequency (*f*_0_) range [8], altered phonation quality [9], and inaccurate and reduced articulation [10–12]. All these changes affect listeners' perceptions, such as perceived softer speech, reduced voice quality, and poor articulation. As a result, impaired intelligibility is very common in PD [13].

Various pharmacological and surgical techniques are now available to help manage the different motor symptoms of patients with PD. One of these surgical techniques is deep brain stimulation of the subthalamic nucleus (STN DBS). Even though it has been demonstrated that STN DBS can drastically reduce motor symptoms and improve patients' quality of life, it has been associated with relatively small changes in dysarthria severity levels and intelligibility. In most studies, the impact of STN DBS on dysarthria was at best mixed and was, more often than not, minor and/or poor [14]. For a review of the impact of STN DBS on speech systems, see [15].

The articulation of speech sounds requires fine motor control. Speech units can be characterized in terms of articulatory gestures (range of movement) or in terms of acoustic distinctiveness. Acoustic distinctiveness refers to the fact that two speech units that are acoustically differentiated are more easily identified by our perceptual system [16]. Using this paradigm, a category of sounds—the vowels—can be acoustically described and differentiated from one another by their acoustic characteristics. The principal acoustic characteristics used to describe vowels are their formants. The first two formants of a vowel, *F*1 and *F*2, are spectral values that allow categorization of the phoneme. *F*1 and *F*2, respectively, serve as indicators of the open-close and front-back position of the articulators (jaw, tongue) during the production of speech [17]. The articulation of vowels is very important for speech intelligibility, and reduced acoustic distinctiveness of vowels has been reported in studies of dysarthric speakers, including people with PD [18]. In a study analyzing different metrics to evaluate the effects of STN DBS on speech production, Weismer et al. [19] suggested that vowel production has promising results regarding articulation measurements. A few studies [20, 21] investigated the changes in vowel production that could occur with STN DBS in PD. However, these studies looked at speech components other than articulation, such as voice quality or speech rate.

Coarticulation is another important acoustic factor for speech intelligibility [22, 23]. Coarticulation can be defined as the influence speech units have on each other in connected speech [24]. Coarticulation effects fall into two categories and are described as anticipatory (“right to left”) or carry-over (“left to right”). Anticipatory coarticulation is generally considered a consequence of motor planning because the positions of the articulators for a given speech gesture are modified in prediction of a following gesture. Carry-over coarticulation, on the other hand, is considered a consequence of articulatory inertia and is influenced by the physical characteristics of the articulators [25]. Initially investigated in normal speakers, more and more studies looked at possible changes in coarticulation patterns in dysarthric speakers [26, 27]. Coarticulation is a marker of speech fine motor control. It can be acoustically measured with different methods, including analyzing the influence of a given context on the articulation of the following phoneme (carry-over coarticulation). In some studies, authors used this type of measurement to characterize coarticulation in normal speakers [28–30]. They showed that, in a consonant-vowel (C-V) sequence, the place of articulation of the consonant modulates the formant values of the vowel. Some studies showed that STN DBS can induce changes in fine motor control during speech production [15]. One could therefore presume that coarticulation could be influenced by STN DBS. However, this hypothesis has not been specifically investigated in any previous studies.

Even though articulatory function is very important for speech intelligibility, particularly for vowels, only a few studies examined the impact of STN DBS on articulation in PD, in terms of range of movement. Moreover, to the best of our knowledge, no studies have investigated the coarticulation changes that could occur with STN DBS. Therefore, the primary objective of this study was to examine the effects of STN DBS on speech articulation in dysarthric speakers with PD, using acoustic measurements of vowels. The second objective was to examine the coarticulation changes with STN DBS, by analyzing the impact of the preceding consonant context on vowel articulation, which represents a measure of carry-over coarticulation.

## 2. Methods

### 2.1. Participants

#### 2.1.1. Demographic Characteristics

The study was approved by the local institutional ethics committee for the safety of human subjects and written informed consent was obtained from all participants. Eight participants (5 women and 3 men) aged 53–72 years, with idiopathic PD diagnosed 9 to 25 years prior to the study, were recruited in an outpatient clinic. All participants were taking antiparkinsonian medications, with L-dopa equivalent dose from 300 to 1500 mg per day, and no changes were made to their medication during the study. All of them were native speakers of Quebec French who had always lived in the province of Quebec. Although no formal hearing evaluation was conducted, all participants were functional in conversation and none reported any hearing impairment. General cognitive functions were measured using the Montreal Cognitive Assessment (MoCA) [31] and no participant fell below the cut-off score according to age and education level [32]. Each participant with PD was also asked to complete the French version of the Voice Handicap Index (VHI) [33], which is one of the most common instruments designed to measure quality of life with respect to dysarthria in PD. Higher values indicate greater voice handicap. Characteristics of each participant are reported in Table 1.

#### 2.1.2. Speech Characteristics

Two speech-language pathologists were asked to reach an agreement on dysarthria diagnosis, dysarthria severity, and acoustic characteristics for each participant during ON-stimulation. Their evaluation was based on audio recordings of each participant reading “La bise et le soleil” [34], a standardized French text commonly used in French phonetic experiments to study normal or pathological speech [35]. This text is considered the French equivalent of the English text* The Rainbow Passage* [36]. Each recording was presented to the two speech-language pathologists independently in a quiet room via open-air speakers. Dysarthria severity ranged from mild to severe, with altered phonation, reduced articulation, and abnormal speech rate being the main acoustic features identified in most of the recordings. All participants were diagnosed with hypokinetic dysarthria, which is consistent with the speech profile generally observed in people with PD [13]. Although the presence of a speech disorder was not a criterion to be eligible for this study, all participants reported speech difficulties.

#### 2.1.3. Deep Brain Stimulation Characteristics

The participants in this study had undergone bilateral DBS of the STN surgery 2 to 5 years prior to the study. All of them had been operated on by the same neurosurgeons (LC and MP) and were regularly followed by the same neurologist (ML). Surgery was done under local anaesthesia and sedation with the CRW stereotactic frame. The day before surgery, patients had high-resolution T2-weighted MRIs (3.0-T unit, Siemens). These images were fused with a T1-Gadolinium (double dose, 1.5-T unit, Siemens) acquired with the localization frame on the day of surgery. Neuronavigation (Stealth System from Medtronic, Inc., Minneapolis, MN) was used to plan the surgery and fuse the images. The target was the STN and was calculated from the mid-commissural point. The indirect coordinates were 3 mm behind the mid-commissural point, 12 mm lateral, and 4 mm inferior. The target was confirmed by microrecording and microstimulation; then a quadripolar lead was implanted (Model 3387, Medtronic, Inc., Minneapolis, MN). Surgery was done on both sides on the same day. One to 3 days later, the neurostimulator was implanted (Activa System, Medtronic, Inc., Minneapolis, MN).

At the time of the study, the electrical parameters of the DBS had been stable for a period of at least 2 months. For 7 of the 8 participants, stimulations were delivered at a frequency of 145–185 Hz and a voltage of 3.0–3.8 V. For one participant (PD1), the stimulations were delivered at a lower frequency (60 Hz) but higher voltage (5.5 V left, 6.0 V right). The configuration of the electrical parameters for each participant was selected and adjusted by their neurologist, based on observed and reported clinical symptoms of MP such as tremor, rigidity, speech difficulty, and dyskinesia. Individual DBS parameters for each participant are reported in Table 2. No postoperative measurements of the electrodes' location were available at the time of recruitment.

### 2.2. Evaluation Sessions

Two evaluation sessions for each participant took place on different days. Participants PD4, PD5, and PD8 were first recorded in the ON-stimulation state and then at least 6 weeks later in the OFF-stimulation state. For the other participants, OFF-stimulation recordings took place first and ON-stimulation recordings were made afterward. This design was chosen to counterbalance the possible habituation effects of the task between the two stimulation conditions. ON-stimulation recordings were made at home with no change to the stimulation settings used in the participant's everyday life. OFF-stimulation recordings were made at the hospital under medical surveillance and took place one hour after the stimulator was turned OFF. All these sessions took place in a quiet room at the same time of the day for each participant to minimize variations in medication cycles. All participants took their antiparkinsonian medication at least one hour prior to the sessions and were in ON-state medication during the recordings. The evaluations were administered by the first author of this paper (VMS) with the collaboration of a graduate student specializing in speech and language disorders in PD.

#### 2.2.1. Neurological Assessment

At both evaluation sessions, the severity of motor symptoms was measured using the motor section of Unified Parkinson's Disease Rating Scale (UPDRS-III) [37]. Disease stage was estimated using the adapted Hoehn and Yahr scale [38]. This neurological assessment was done for each participant to document the impact of their antiparkinsonian medication as well as the long-term effects of STN DBS on their motor symptoms.

#### 2.2.2. Speech Assessment

All recordings were made using a Shure 510A head-mounted microphone and a Zoom H4n audio recorder at a sampling rate of 44.1 kHz. Mouth-to-microphone distance was approximately 4 to 8 cm for each participant but remained constant throughout the recording session. Vowel articulation was measured with a reading-aloud task of spoken “consonant-vowel-consonant-vowel” (CVCV) tokens, with the target vowels /i/, /u/, and /a/ and the consonants /p/, /t/, /k/, /b/, /d/, and /g/ (plosives) and /f/, /s/, and /*ʃ*/ (“ch”) (fricatives). The use of these three vowels provided a way to measure maximum vowel space acoustically occupied by each participant. These consonants were selected because they represent a variety of accepted phonemic contexts in French. The plosive contexts also enabled us to investigate vowel articulation as a function of the preceding consonantal context (/p/ and /b/ for labial context, /t/ and /d/ for alveolar context, and /k/ and /g/ for velar context), which represents a measure of coarticulation. These tokens were embedded in the carrier phrase “*Je pense CVCV cette fois*” (“I think CVCV this time”) in order to standardize the prosody and accentuation of productions. The task was repeated twice in each recording session (ON- and OFF-stimulation) and each individual token occurred twice in each repetition. A total of 108 productions (9 consonants × 3 vowels × 2 token occurrences × 2 task repetitions) were thus recorded per participant per stimulation condition. The order of presentation of the tokens was randomized but this sequence remained the same between all participants and throughout all recording sessions.

### 2.3. Acoustic Analyses

Acoustic analyses were done by a trained phonetician using Praat software v5.3.30 [39] running on Windows OS. Acoustic segmentations were conducted using different visual criteria on a spectrogram and oscillogram. Multiple scripting procedures were implemented in the analyses when no manual intervention was required.

#### 2.3.1. General Vowel Articulation

Vowel articulation was measured by analyzing *F*1 and *F*2 formant frequencies of /i/, /u/, and /a/ of the last vowel of the CVCV tokens in a 500-ms analysis window. Vowel duration was also measured for covariance analyses. Vowel onset was first determined by the appearance of stable formant frequencies on the spectrogram, and the offset was determined by the last glottal pulse visible on the oscillogram. With these values, two variables were calculated. The first variable is the* acoustic vowel space* (AVS), which is the surface of the triangle formed by the *F*1 and *F*2 formant values of the vowels /i/, /u/, and /a/. Higher AVS values correspond to* increased* vowel articulation. This variable is calculated using the following formula:

(1) $$\begin{array}{c} \text{AVS}=\left|\frac{F2\text{i}\left(F1\text{u}-F1\text{a}\right)+F2\text{u}\left(F1\text{a}-F1\text{i}\right)+F2\text{a}\left(F1\text{i}-F1\text{u}\right)}{2}\right|. \end{array}$$

The second variable is the* formant centralization ratio* (FCR), which is a coefficient that represents the magnitude of centralization of the formants *F*1 and *F*2 for vowels /i/, /u/, and /a/. This metric was developed by Sapir and colleagues [10] and has been used in other studies on vowel articulation in PD. Higher FCR values represent higher formant centralization and consequently* reduced* vowel articulation. This variable is calculated using the following formula:

(2) $$\begin{array}{c} \text{FCR}=\frac{F2\text{u}+F2\text{a}+F1\text{i}+F1\text{u}}{F2\text{i}+F1\text{a}}. \end{array}$$

#### 2.3.2. Vowel Articulation by Consonant Context

Vowel articulation was also measured as a function of the preceding consonant context by measuring *F*1 and *F*2 only for vowels in labial (/p/ and /b/), alveolar (/t/ and /d/), and velar (/k/ and /g/) contexts and then calculating AVS for each context representing a place of articulation. The fricative contexts were excluded from these analyses because their place of articulation is not comparable to those of the plosive contexts. Segmentation criteria for this variable were the same as those used for general vowel articulation.

### 2.4. Reliability of Acoustic Data

Due to the nature of the speech samples acquired in this study, the formant detection algorithms used by the* Praat* software can produce outlier artefacts [40]. To minimize the number of these erroneous formant values in our data pool, a statistical multivariate outlier detection procedure was applied to the measured *F*1 and *F*2 frequencies for individual vowels and for each participant. Using leverage values, standardized residual scores, and influence factors [41], data differentiated at *P* < 0.01 were excluded from further analysis. With this procedure, 23 data points (1.3%) of the entire data pool were rejected. Also, due to the recording methods and participants' fluctuating voice quality (e.g., oversaturation of the microphone, voice breaks, and no discernible glottal pulse), 41 data points (2.4%) could not be analyzed. Finally, one participant (PD2) was not able to complete the second repetition of the task during OFF-stimulation due to fatigue. Of the 54 tokens in the second repetition, 43 are missing.

## 3. Results

### 3.1. Data Pooling

Because the speech task was administered twice per experimental condition, preliminary analyses were made to verify differences between the two repetitions under both conditions. On all variables, no statistical difference was found between both repetitions during ON- and OFF-stimulation. Therefore, data obtained in both repetitions during ON- and OFF-stimulation were pooled for subsequent analyses, without considering the occurrence of the repetition.

### 3.2. Statistical Analyses

The effect of the electrical stimulation condition (ON-stimulation versus OFF-stimulation) on each variable under study was analyzed using a mixed model analysis of variance (ANOVA). Repeated measure factor (occurrence of the repetition) was entered in the model and was based on an unstructured covariance matrix, which allows for unequal variance between each repetition [41]. Participants were entered in the model as a random factor and were based on a scaled identity covariance matrix. Finally, all dependent variables were analyzed using the stimulation condition (ON versus OFF) as a fixed factor. Subsequent analyses were conducted by entering the consonantal context (labial versus alveolar versus velar) as another fixed factor. All statistical analyses were conducted using* SPSS* v. 20 [42]. This procedure follows the guidelines suggested in [43] for phonetic research in order to avoid statistical problems like pseudo-replication of data.

### 3.3. Neurological Assessment

Table 3 reports means and standard deviations of the total UPDRS-III score and Hoehn and Yahr stage in both OFF- and ON-stimulation conditions. Statistical analyses indicate a significantly lower total UPDRS-III score during ON-stimulation, which indicates that the electrical stimulations of DBS reduce the severity of motor symptoms. For the Hoehn and Yahr stage, a statistical tendency (*P* = 0.07) was obtained (average ON score: 2.26; average OFF score: 2.63). The score on the single speech item (item 18) of the UPDRS-III was also analyzed separately. Scoring for this item reflects the degree to which speech is impaired, that is, considered not impaired (0), mildly impaired (1), moderately impaired (2), severely impaired (3), or mostly unintelligible (4). Analyses indicate that the average score on this item was slightly lower during ON-stimulation (average ON score: 2.06; average OFF score: 2.19), but the difference failed to reach statistical significance. In both conditions, none of the participants showed signs of dyskinesia or reported other side effects.

### 3.4. General Vowel Articulation

**Figure 1:**
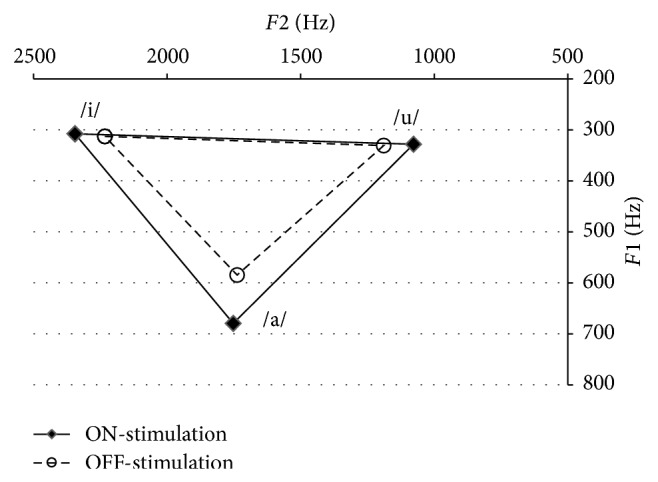
Acoustic space of average *F*1 and *F*2 values of the vowels /i/, /u/, and /a/ during OFF-stimulation and ON-stimulation.

Table 4 reports means and standard deviations for *F*1 and *F*2 formant frequencies (Hz) for the vowels /i/, /u/, and /a/ during OFF-stimulation and ON-stimulation. Descriptive data for *F*1/*F*2 AVS (Hz^2^), FCR, and vowel duration (msec) are also reported for the OFF and ON conditions. Statistical analyses indicate a significant increase in AVS (+75723 Hz^2^) and decrease in FCR (−0.098) in the ON-stimulation condition. No significant change was found in vowel duration. These results indicate that maximum vowel articulation increased with the electrical stimulations and that this change was not related to the duration of the vowels.  Figure 1 displays the average *F*1 and *F*2 values for /i/, /u/, and /a/ during OFF-stimulation and ON-stimulation in a standard *F*2/*F*1 acoustic space. Visual analysis of the maximum acoustic space indicates that the main changes in vowel articulation that occur with electrical stimulations are on *F*2 for /i/ and /u/ and on *F*1 for /a/.

### 3.5. Vowel Articulation by Consonant Context

**Figure 2:**
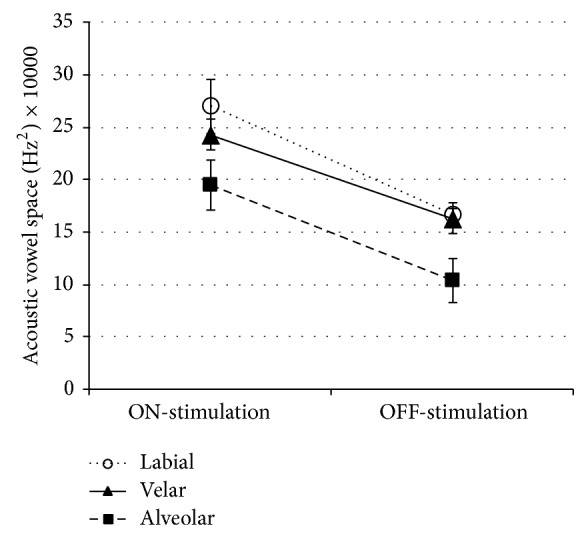
Acoustic vowel space values for vowels in labial, velar, and alveolar context in both stimulation conditions.

Figure 2 displays acoustic vowel space values (Hz^2^) calculated from *F*1 and *F*2 formant frequencies of the vowels /i/, /u/, and /a/ produced in labial, alveolar, and velar consonant contexts during OFF-stimulation and ON-stimulation. Statistical analyses for each context indicate a significant effect of the stimulation condition for each consonant context, with an increase in vowel articulation in the ON-stimulation condition: labial context: *F*(1; 6.8) = 19.71, *P* < 0.01; velar context: *F*(1; 6.9) = 13.46, *P* < 0.01; alveolar context: *F*(1; 7.0) = 32.31, *P* < 0.01. Furthermore, a mixed model ANOVA analysis was performed on data from all contexts with stimulation (OFF versus ON) and consonant context (labial versus alveolar versus velar) entered as fixed effects. A significant effect of the stimulation was found: *F*(1; 943.3) = 22.55, *P* < 0.000, with an increase in vowel articulation in the ON-stimulation condition. A significant effect of the consonant context was also found: *F*(2; 38.0) = 31.97, *P* < 0.000, where vowel articulation increased following the contexts: alveolar < velar < labial. On the other hand, no statistical interaction effect between the stimulation condition and consonant context was found: *F*(2; 38.0) = 0.621, *P* > 0.05. These results indicate that vowel articulation is influenced by the preceding consonant context but that this effect is not modulated by DBS, whether the stimulation is OFF or ON.

## 4. Discussion

This study reports results regarding the impact of the electrical stimulations of bilateral STN DBS on vowel articulation in 8 individuals with PD. With respect to motor symptoms, when STN DBS was turned OFF, the severity of motor symptoms, measured with the UPDRS-III, was significantly greater and the Hoehn and Yahr stage marginally increased. Other studies investigating the impact of STN DBS on motor symptoms in PD concluded that bilateral implantation induces greater improvement in motor symptoms in people presenting more severe motor symptoms [44]. Our results concerning motor symptoms are consistent with these previous studies. It is also important to note that none of the participants reported side effects directly related to STN DBS in our study. Specifically, many side effects (such as stimulation-induced dyskinesia, stimulation-induced hypotonia or apraxia of eyelid opening) are frequently associated with nonoptimal stimulation settings [45]. The absence of such side effects, as well as the reduction in motor symptoms measured by the UPDRS-III, suggests that the electrical settings were optimally configured for each participant in our study.

With respect to speech, the impact of STN DBS on vowel articulation was analyzed using the first two formants of the target vowels /i/, /u/, and /a/ and then by calculating two acoustic factors: acoustic vowel space and formant centralization ratio. These two measures serve as acoustic markers of vowel articulation and represent the range of articulatory movement during the production of vowels for each participant. Our results indicate that STN DBS increased vowel articulation while vowel duration remained unchanged. Shorter vowels are usually related to vocalic undershoot in many studies [46]. Our result is therefore important since it can be assumed that the change in articulation we observed with STN DBS is due to a change in the articulatory range of movement* per se*, rather than a change in speech rate or vowel duration.

Further analyses of formant values for each vowel enable a more precise description of the acoustic changes that occur with STN DBS and, by extension, its impact on speech motor control. For /i/ and /u/, the main changes with STN DBS occurred on *F*2 (increase for /i/ and decrease for /u/) while *F*1 remained stable. *F*1 is usually associated with the width of the resonance cavity (aperture) while *F*2 represents the length of the cavity (front-back distinction). Therefore, our results indicate that the electrical stimulations of STN DBS improve the range of anteroposterior movement of the tongue dorsum. For the vowel /a/, STN DBS led to an increase on *F*1 while *F*2 remained stable. *F*1 is the acoustic factor that represents vowel aperture, controlled by the jaw. Therefore, this result indicates that STN DBS may improve the range of movement in the jaw opening. Among the three vowels we analyzed, /a/ showed the broader change with STN DBS. This result indicates that different articulatory gestures in speech production may be differently affected by STN DBS. Studies investigating speech motor control with STN DBS in PD using articulatory measurements are scarce. Further studies should examine the impact of STN DBS in PD on the distinct jaw/tongue/lips articulatory processes, in terms of range, target, and velocity movements.

In the present study, we observed significant changes in vowel articulation as a function of the preceding consonant context, which represents an acoustic marker of coarticulation. More specifically, we showed that articulation was significantly reduced when vowels were produced in an alveolar context, as compared to velar and labial contexts. Even though the consonant context influences vowel articulation, this coarticulatory phenomenon did not vary as a function of the STN DBS in our study. To our knowledge, our study is the first to look at the impact of STN DBS on coarticulation. The acoustic metric used in this study investigated the impact of the preceding consonantal context on vowel articulation in its stable portion, which is a measure of carry-over coarticulation. Even though our results indicate that this type of coarticulation is not sensitive to STN DBS, the impact of the stimulation on speech coarticulation should be explored in future studies because (1) it has been shown to be altered in some studies on dysarthria in PD [26] and (2) it is essential to speech intelligibility [47]. In this regard, future studies could also investigate the impact of STN DBS on anticipatory coarticulation because it is a marker of motor programming [25] and implies mechanisms complementary to those implied in carry-over coarticulation.

Studies investigating articulatory changes with STN DBS in PD are almost nonexistent. In a preliminary report, Dromey and Bjarnason [48] studied the impact of electrical stimulations on speech and language characteristics in people with PD, including vowel articulation. Of their six patients, two showed increased vowel articulation in the ON-stimulation condition, three showed decreased articulation, and one did not vary. However, the authors did not specify the individual electrical parameters of the stimulations. Stimulation frequency and voltage were associated with other altered speech systems in some studies [49] so that interpretation of their mixed results is difficult. The lateralization of the stimulations may also have an impact on articulation. Wang and colleagues [50] analyzed the impact of left- versus right-side STN DBS on different speech mechanisms, including articulation accuracy. They concluded that left-side stimulation altered articulation accuracy while it remained unchanged or even improved with right-side stimulation. In our study, all the patients had undergone bilateral STN DBS, which may have better effects on speech than unilateral stimulation, particularly compared to left-side stimulation.

The intrinsic characteristic of the pathology (idiopathic PD versus young-onset PD, severity of the motor symptoms) is another factor that may account for the differing results. In our study, all patients were diagnosed with idiopathic PD with age at onset greater than 40 years, which may explain why our results are homogeneous. Surprisingly, to our knowledge, speech characteristics and dysarthria profiles in PD in regard to onset of the disease have not been investigated. Future studies should examine this aspect more closely.

### 4.1. Limitations of the Study

Some limitations must be taken into consideration when interpreting the findings of this study. First, this investigation was conducted with only a small number of participants. Generalization of these results must therefore be viewed with caution and the large inter- and intravariability in speech disorders and clinical presentation in people with PD must be kept in mind. Another limitation of this study is that our speech measurements were taken only in reading tasks, which may cast doubt on the naturalness of the speech. This type of limitation is commonly recognized in phonetic studies [10, 51] but it is a methodological choice made to control the phonemic, syntactic, and prosodic contexts around the target sounds.

With the exception of item 18 of the UPDRS-III (which did not vary significantly with STN DBS), speech intelligibility was not formally assessed here. Even though vowel articulation is a strong factor associated with intelligibility, the impact of the changes in vowel articulation on overall speech intelligibility must be viewed with caution because additional acoustic factors are responsible for reduced intelligibility in PD, such as phonation or prosodic disturbances.

## 5. Conclusions

This study is one of the first to investigate changes in vowel articulation and coarticulation that occur with STN DBS in PD in terms of maximum range. Using acoustic measurements, we found that bilateral STN DBS improves articulation in terms of anteroposterior tongue movements and jaw opening during speech production. Coarticulation did not change as a function of the stimulations. Previous studies that investigated the impact of STN DBS on different speech systems in PD had mixed results. Differences in stimulation parameters and configuration criteria may explain these differences. According to our results, dysarthria is a symptom of PD that is sensitive to STN DBS. Therefore, speech quality should also be considered when optimizing stimulation parameters for patients. When optimized, stimulation should not negatively affect speech and could, in fact, improve some aspects, such as articulation.

## Figures and Tables

**Table 1:** Characteristics of participants.

Participant	Age	Sex	Education	MoCA^a^	Years post-Dx	L-Dopa dose	VHI score^b^
PD1	66	M	9	25	25	1500 mg	83
PD2	66	F	11	26	16	600 mg	34
PD3	71	M	6	25	11	500 mg	49
PD4	72	F	19	26	19	300 mg	56
PD5	53	F	11	29	12	300 mg	34
PD6	65	F	12	27	14	500 mg	51
PD7	72	F	19	25	19	500 mg	48
PD8	69	M	13	24	9	300 mg	42

Notes: ^a^MoCA = Montreal Cognitive Assessment.

^b^VHI = Voice Handicap Index.

Years post-DX = Years after the diagnosis has been made.

**Table 2:** Individual DBS parameters.

Participant	Years post-op	Frequency (Hz)		Voltage (V)		Pulse width (*µ*s)		Electrode configuration (“0” = off, “+” = anode, “−” = cathode)									
		Left	Right	Left	Right	Left	Right	Left contacts					Right contacts				
								0	1	2	3	IPGc	0	1	2	3	IPGc
PD1	4	60	60	5.5	6.0	60	60	0	0	0	−	+	0	0	0	−	+
PD2	4	145	145	3.5	3.8	90	90	+	0	−	−	0	+	0	−	−	0
PD3	4	185	185	3.3	3.3	60	60	0	0	−	−	+	0	0	−	−	+
PD4	5	185	185	3.5	3.5	90	90	0	0	−	−	+	0	0	−	−	+
PD5	4	185	185	3.7	3.7	90	90	+	0	−	−	0	+	0	−	−	0
PD6	3	145	145	3.5	3.5	60	60	0	+	−	0	0	0	0	−	+	0
PD7	3	180	180	3.0	3.7	60	60	0	0	−	−	+	0	0	−	0	+
PD8	2	180	180	3.8	3.8	60	60	0	0	−	−	+	0	0	−	−	+

Notes: IPGc = Internalized Pulse Generator Case.

**Table 3:** Scores on motor subscale of the UPDRS (no symptoms = 0), Hoehn and Yahr (H&Y, mild = 1) staging during ON- and OFF-stimulation: means (standard deviations), *t* and *P* values.

	UPDRS-III (total)	H&Y^a^	UPDRS-III^b^ (speech item)
OFF-stimulation	48.3	2.63	2.19
	(16.7)	(0.74)	(0.88)
ON-stimulation	29.7	2.26	2.06
	(9.18)	(1.04)	(0.62)
*t*	4.39	2.05	0.68
*P*	<0.01∗	0.07	>0.05

∗Significant effect.

^a^H&Y = Hoehn and Yahr.

^b^UPDRS-III = Unified Parkinson's Disease Rating Scale.

**Table 4:** Formant frequencies, acoustic vowel space, formant centralization ratio, and vowel duration at OFF- and ON-stimulation: means (standard deviations), *F* and *P* values.

	/i/		/u/		/a/		Acoustic vowel space (Hz^2^)	Formant centralization ratio	Vowel duration (ms)
	*F*1 (Hz)	*F*2 (Hz)	*F*1 (Hz)	*F*2 (Hz)	*F*1 (Hz)	*F*2 (Hz)			
OFF-stimulation	311.5	2233.1	329.2	1186.6	583.3	1736.3	152036	1.240	130.2
	(54.4)	(219.0)	(66.6)	(283.1)	(127.6)	(263.5)	(14251)	(0.035)	(8.8)
ON-stimulation	307.6	2345.7	328.5	1077.0	680.1	1753.0	227759	1.142	138.4
	(45.1)	(264.4)	(61.3)	(250.1)	(91.1)	(268.0)	(17905)	(0.031)	(10.5)

						Change	+75723	−0.098	+8.2
							(11504)	(0.20)	(5.1)
						*F*	43.327	24.482	2.665
						*P*	<0.000∗	<0.01∗	>0.05

∗Significant effect.
